# Target localization of 3D versus 4D cone beam computed tomography in lipiodol-guided stereotactic radiotherapy of hepatocellular carcinomas

**DOI:** 10.1371/journal.pone.0174929

**Published:** 2017-04-06

**Authors:** Mark Chan, Chi Leung Chiang, Venus Lee, Steven Cheung, Ronnie Leung, Matthew Wong, Frankle Lee, Oliver Blanck

**Affiliations:** 1 Department of Radiation Oncology, University Medical Center Schleswig–Holstein, Kiel, Germany; 2 Department of Clinical Oncology, Tuen Mun Hospital, Hong Kong (S.A.R); 3 Department of Clinical Oncology, The University of Hong Kong, Hong Kong (S.A.R); 4 Department of Clinical Oncology, University of Hong Kong-Shenzhen Hospital, China; North Shore Long Island Jewish Health System, UNITED STATES

## Abstract

**Background:**

Aim of this study was to comparatively evaluate the accuracy of respiration–correlated (4D) and uncorrelated (3D) cone beam computed tomography (CBCT) in localizing lipiodolized hepatocellular carcinomas during stereotactic body radiotherapy (SBRT).

**Methods:**

4D–CBCT scans of eighteen HCCs were acquired during free–breathing SBRT following trans–arterial chemo–embolization (TACE) with lipiodol. Approximately 1320 x–ray projections per 4D–CBCT were collected and phase–sorted into ten bins. A 4D registration workflow was followed to register the reconstructed time–weighted average CBCT with the planning mid–ventilation (MidV) CT by an initial bone registration of the vertebrae and then tissue registration of the lipiodol. For comparison, projections of each 4D–CBCT were combined to synthesize 3D–CBCT without phase–sorting. Using the lipiodolized tumor, uncertainties of the treatment setup estimated from the absolute and relative lipiodol position to bone were analyzed separately for 4D– and 3D–CBCT.

**Results:**

Qualitatively, 3D–CBCT showed better lipiodol contrast than 4D–CBCT primarily because of a tenfold increase of projections used for reconstruction. Motion artifact was observed to subside in 4D–CBCT compared to 3D–CBCT. Group mean, systematic and random errors estimated from 4D– and 3D–CBCT agreed to within 1 mm in the cranio–caudal (CC) and 0.5 mm in the anterior–posterior (AP) and left–right (LR) directions. Systematic and random errors are largest in the CC direction, amounting to 4.7 mm and 3.7 mm from 3D–CBCT and 5.6 mm and 3.8 mm from 4D–CBCT, respectively. Safety margin calculated from 3D–CBCT and 4D–CBCT differed by 2.1, 0.1 and 0.0 mm in the CC, AP, and LR directions.

**Conclusions:**

3D–CBCT is an adequate alternative to 4D–CBCT when lipoid is used for localizing HCC during free–breathing SBRT. Similar margins are anticipated with 3D– and 4D–CBCT.

## Introduction

Trans–arterial chemoembolization (TACE) is a common first line treatment for patients with unresectable hepatocellular carcinomas (HCC). However, the tumor response after TACE alone has been frequently incomplete even for repetitive treatments. By combining TACE with hypofractioned stereotactic body radiotherapy (SBRT), improved tumor response and overall survival have been achieved [1,2,3]. One advantage of sequencing TACE prior to radiotherapy is that the potential tumor shrinkage after TACE may allow the radiation field to be reduced, permitting higher tumor doses and/or lower risk of toxicity such as radiation–induced liver disease (RILD). In addition, the lipiodolized tumor after TACE can be exploited as a natural contrast medium for direct target localization in the subsequent SBRT to minimize geometric uncertainties from differential tumor position and/or motion of implanted fiducial markers or diaphragm frequently used as surrogates [4,5,6].

Despite the potential of lipiodol contrast, motion mitigation during image guidance (IG) using on–board cone beam CT (CBCT) is important to reduce the uncertainty of ambiguous target definition when tumor at different positions over the respiratory cycles is compressed onto a single 3D image during reconstruction. As indicated by Sweeney *et al*., motion blurring in free–breathing CBCT can impact the target localization and increase the inter–observer's variability in thoracic SBRT [7]. Single or multiple breath–hold CBCT can effectively minimize the motion–related artifacts hence potentially improved tumor localization accuracy but these IG–SBRT approaches were not tolerable to all HCC patients and increased substantially the duty cycle of imaging and / or treatment delivery [8,9].

Recently, the potential of using four–dimensional (4D)–CBCT reconstructions to reduce motion blurring artifacts has been investigated in SBRT [7,10,11]. Unlike its 3D counterpart, projections data in 4D reconstruction are sorted into bins, each of which produces a three–dimensional (3D) image at a specific time point along the breathing cycle [12]. Such 4D–CBCT reconstruction scheme is also important for completing the implementation of parallel 4D imaging to extract the tumor motion and the time–weighted average (MidP) CBCT images for tumor localization during treatment planning and delivery. By planning and targeting the tumor at the MidP treatment strategy a safety margin that is slightly larger than that for the breath–hold approach can be achieved for free–breathing treatment suitable for all patients [13]. For free–breathing SBRT, Velec *et al*.[14] has demonstrated a potential reduction of gross tumor volume (GTV) to planning target volume (PTV) by 34 ± 7% using the MidP approach compared to the internal target volume–based PTV, which translated into 66 ± 38 cc of normal liver tissues for better dose sparing and potential dose escalation.

Previous comparative studies of clinical accuracy of 3D and 4D–CBCT have largely focused on thoracic lesion [7,10]. Zhang *et al*. demonstrated for two liver cases that motion–compensated 4D–CBCT can further reduce motion blurring and streak artifacts due to sparse projection data and hence resulting in improved visibility of implanted fiducials and tissue contrast between anatomical structures [15]. Yet, their study was unable to directly localize the liver tumors and included too few cases to allow evaluation of the clinical impacts. On the other hand, improvement of HCC localization accuracy of 4D–CBCT under lipiodol–contrast guidance was evidenced by Chan *et al*.[5], showing a possible cranio–caudal margin reduction by up to 11.7 mm that was required to compensate for uncertainty from diaphragm surrogate. Nonetheless, the relative benefit of clinical implementation of 4D–CBCT to 3D–CBCT to improve the accuracy of localizing lipiodolized HCC during free–breathing SBRT remains unknown. The purpose of this study was to validate the clinical usefulness and the impact of 4D–CBCT in comparison to 3D–CBCT on target localization by retrospectively reconstructing CBCT images with and without respiratory correlation. We aimed to (1) quantify the potential deviations of 3D / 4D–CBCT treatment position of the lipiodolized tumor, (2) to investigate the factors that may be predictive of the observed deviations, and (3) to evaluate the safety margin necessary to account for the discrepancy between 3D– and 4D–CBCT imaging in free–breathing SBRT.

## Methods and materials

### Ethics statement

This study was approved by the local ethics committee of the New Territories West Cluster of Hospital Authority Hong Kong. The need of written informed consent from the participants was waived because this study was not treatment-based. For protection of patient confidentiality, the patient information was anonymized.

### Patient eligibility and treatment

Eighteen patients with non–resectable HCC were de–identified and included in this retrospective analysis. The tumor volume ranged from 10 to 1100 cc (median = 186 cc). One to two cycles of first–line TACE was given to these patients one week prior to 4D–CT simulation [16]. Individualized dose fractionation of either 4 Gy for 6–10 fractions or 6–9 Gy for 6 fractions was prescribed according to estimated risk of RILD [17,18]. In all cases, SBRT plans were developed with volumetric–modulated arc (VMAT) technique and delivered under free–breathing condition three to four weeks after TACE.

### 4D–CT simulation and treatment planning

4D–CT imaging was performed in helical mode with 2 mm axial slices on a Philips Brilliance BigBore scanner (Philips Medical Systems, Cleveland, USA). A phase–based approach was used to sort the 4D–CT raw data into ten series of 3D images each sharing equal time percentage of the breathing cycle. At our institution, treatment planning was based on the mid–ventilation (MidV) planning target volume (PTV) concept and followed the method described in Ref. [19] for motion extraction from the 4D series. As discussed by Wolthaus *et al*. the tumor at the mid–ventilation may not correspond to its time–weighted average position (MidP) due to motion hystersis [20]. The mean tumor vector errors of the MidV relative to the time–weighted average tumor position (MidP), however, were generally less than the variability due to physiologic changes reported to be within 1.0 ± 0.5 mm [14].

For all patients, the lipiodol retention on the MidV images was used as contrast medium to assist delineation of the gross tumor volume (GTV) and to provide the tumor motion trajectory over the breathing cycle. The tumor motion was then used to formulate the individual safety margin in the *x*, *y*, and *z* directions corresponding to the left–right (LR), cranio–caudal (CC) and antero–posterior (AP) directions, respectively. The safety margin to ensure dose coverage to 90% GTV was calculated by the van Herk’s recipe [12] $M=2.5\Sigma +\beta \sqrt{\left(\sigma^{2}+\sigma_{p}^{2}\right)}-\beta \cdot \sigma_{p}$, where *Σ* and *σ* are the components of systematic and random errors including tumor delineation, 4D–CT/CBCT registration, intra–fractional baseline drift, *β* = 1.03 for the 85% isodose level (IDL) and *σ*_*p*_ = 3.2 mm for penumbra in water. For 4D–CT/CBCT registration, both *Σ* and *σ* were corrected to effective *Σ*_*eff*_ and *σ*_*eff*_ accounting for the finite number of SBRT fractions [21], with averaged eight fractions in our study cohort, prior to margin calculation.

Volumetric–modulated arc radiotherapy was individually optimized with SmartArc (Pinnacle^3^ v.9.2, Philips Radiation Oncology Systems, Fitchburg, USA) using 1–2 partial arcs and mixed energies of 6 and 10 MV.

### CBCT image-guidance correction protocol

Daily pre–treatment CBCT verification was performed using the 4D Symmetry protocol which acquired approximately 1320 projection images at 5.5 frames per second over 200° in 4 minutes. The projection image data were sorted by the Amsterdam shroud method [12], and subsequently reconstructed into a series of 3D–CBCT image datasets corresponding to ten phase positions along the breathing cycle at equal distance plus another at the MidP. This study followed the 4D correction protocol described by Sonke *et al*. [22]. It involved a two–stage registration procedure and started with an automatic rigid bone registration between the reference MidV–planning images and the verification MidP–CBCT images over a region–of–interest around the vertebrae. This step yielded three rotational and three translational errors and because the following 4D registration did not consider rotations, the patient was repositioned whenever the rotation error in any direction was > 1.5°. For final positioning, a 4D registration was performed via local registrations over the planning lipiodol GTV contour with isotropic 5 mm expansion with each phase of the 4D–CBCT scan. The resulting displacement of the MidP from the treatment iso–center was then applied to correct the treatment couch. At this point, the registration was visually inspected, and necessary adjustment was made by an experienced clinician.

Image–guidance correction was further simulated for the 3D–CBCT setup. For this purpose, all projection images were combined without correlating with the respiratory trace to reconstruct a single 3D–CBCT image dataset. Following our institutional protocol of 3D–CBCT setup verification, a soft–tissue registration considering both translations and rotations was performed with a 3D region–of–interest (ROI) including the lipiodol contour. Rotational errors were corrected in the same manner as described in the 4D registration protocol, i.e. using the spinal vertebra. Finally, necessary adjustments were made manually based on the visual inspection of the lipiodol by the same clinician.

### Quantitative assessment of the lipiodol position on 3D– / 4D–CBCT

For inclusion of the lipiodol retention in the analysis, either homogeneous accumulation or partial defect in both the simulation 4D–CT and the setup verification 4D–CBCT scans was considered as acceptable. A total of 105 scans were analyzed, 4–7 scans for each patient including the first and last scans, and other 2–5 scans in between. Inter–fractional changes of absolute and relative lipiodol position to the bony vertebrae were analyzed from the results of the 3D– and 4D–registration of each scan as the difference of the lipiodol centroid position on the 3D– and 4D–CBCT at MidP, compared with that at MidV on the planning 4D–CT. The agreement of inter–fractional change of the lipiodol position between 3D– and 4D–CBCT correction was assessed by Pearson’s correlation coefficient (*r*).

### Impact of 4D–CBCT for target localization and predictive factors

The 4D–CBCT was taken as the reference treatment setup in our online correction protocol. The difference between the clinical 4D–CBCT and the retrospective reconstructed 3D–CBCT registrations provides the tumor prediction error **e** of a 3D–CBCT guided treatment setup. For each patient, **e** was calculated as the mean prediction error ${\bar{e}}_{x/y/z}$ of all scans for the LR/CC/AP directions. Because of the lobe dependence of liver tumor motion [6], we measured the distance from the tumor to the diaphragm (*D*) to investigate its correlation with $\left|{\bar{e}}_{x/y/z}\right|$. *D* was measured as the distance from the dome of the diaphragm to the last seen GTV contour in the cranial direction on the 4D–CT simulation scan. Other predictive factors of $\left|{\bar{e}}_{x/y/z}\right|$ which included the tumor motion range in the planning 4D–CT and the tumor size were also studied.

### Statistical analysis

The individual mean (μ) and standard deviation (SD) of the inter–fractional changes of the lipiodol position were estimated separately for 3D– and 4D–CBCT. Group means (GM) were calculated by averaging the individual patient means. The pooled population effective systematic (*Σ*_*eff*_) and random errors (*σ*_*eff*_) were also calculated. Population statistics were also obtained for the tumor prediction error for estimating the margin with the 3D–CBCT based correction protocol.

The Pearson’s correlation coefficient was used to quantify the correlation of the lipiodol position between 3D and 4D–CBCT and the difference of the absolute group means from zero was evaluated by one sample *t–*test. Differences of the tumor prediction errors and their variability (1 SD) were tested by one–way ANOVA. All tests were performed in SPSS (IBM, USA).

## Results

### Quantitative assessment of the lipiodol position on 3D– / 4D–CBCT

**Fig 1:**
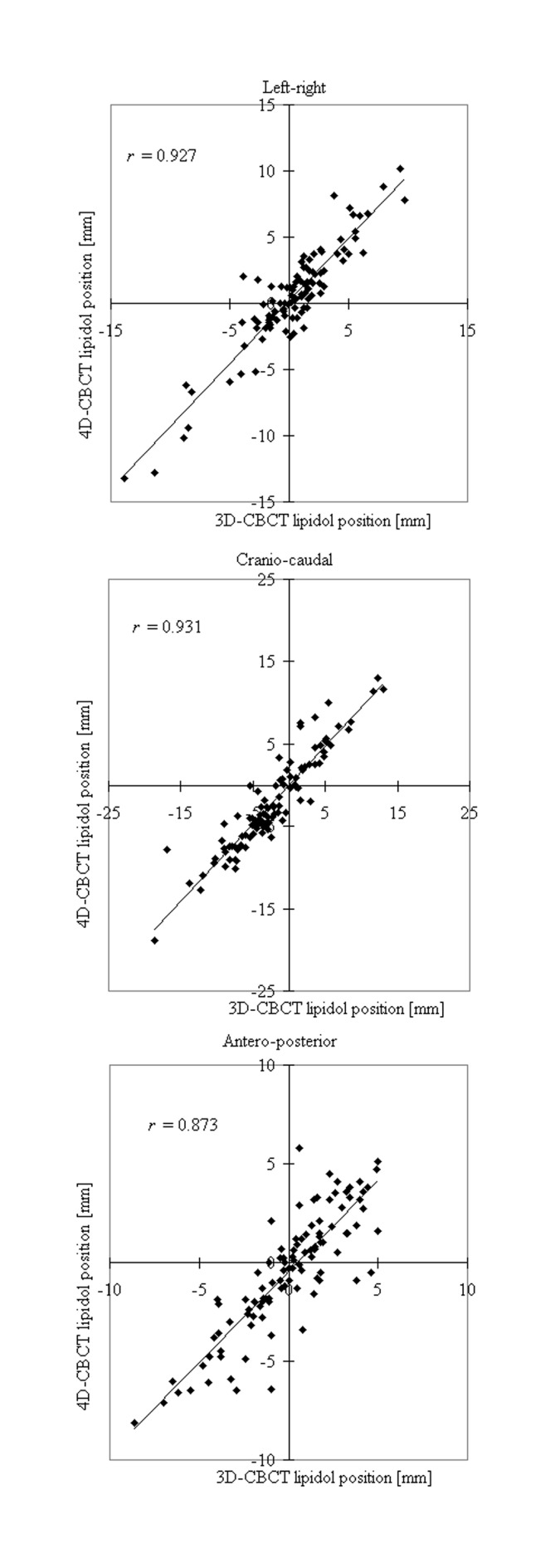
Relationships of lipiodol position in 3D– and 4D–CBCT for all 105 fractions of all 18 patients in the three cardinal directions. Solid lines are the linear fits. Corresponding Pearson’s correlation coefficients (*r*) are also indicated.

**Fig 2:**
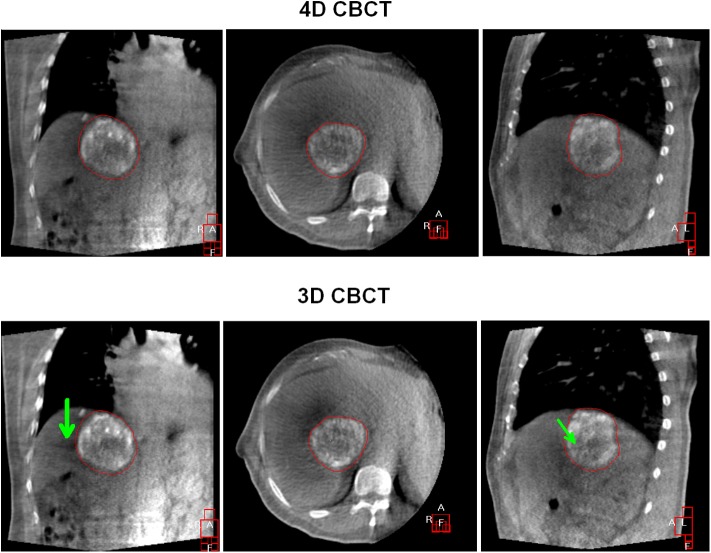
Registration results are highly similar between 3D– and 4D–CBCT (displayed in same window / level). Effect of motion artifact is more pronounced on the 3D–CBCT images (bottom) than on the time–weighted average 4D–CBCT images (top), as indicated by the green arrows. The lipiodol contrast, however, is visualized brighter on 3D–CBCT than on 4D–CBCT due to a tenfold increase of projection data.

Relationships of the absolute lipiodol centroid position between 3D and 4D–CBCT were statistically significant in all directions (Fig 1), with Pearson’s correlation coefficients (*r*) of 0.927 (LR; *p* < 0.01), 0.931 (CC; *p* < 0.01), and 0.873 (AP; p < 0.01). On average, the lipiodol was clearly visualized on both 3D– and 4D–CBCT scans, resulting in similar absolute lipiodol position (example presented in Fig 2).

The quantitative assessment of the 4D–CBCT based treatment setup and the tumor prediction error using 4D–CBCT as the reference are listed in Table 1. Both systematic and random errors estimated from 3D– and 4D–CBCT are within 0.2 mm for all directions, except for the systematic error in the CC direction which was limited to within 1 mm. Statistically, the group means did not differ between the absolute lipiodol position on the 3D– and 4D–CBCT for all directions. They also insignificantly differed from zero except for the CC direction where both 3D– and 4D–CBCT positioning showed an average offset of 3 and 2 mm towards the caudal direction with respect to the planning MidV position. Errors of lipiodol position relative to the bony vertebrae (i.e., the tumor baseline drift) are also given in Table 1.

**Table 1:** Population statistics of the absolute / relative positions of the lipiodol with respect to the vertebra in 3D– and 4D–CBCT, and their difference of absolute position in terms of group means (*GM*), effective systematic (*Σ*_*eff*_), and random (*σ*_*eff*_) errors.

	Left–right (mm)	Cranio–caudal (mm)	Antero–posterior (mm)
3D–CBCT	absolute / relative	absolute / relative	absolute / relative
*GM*	0.2 / 0.2	-2.6 / -1.7	-0.1 / -0.9
*Σ*_*eff*_	3.4 / 2.5	4.7 / 3.7	2.3 / 2.3
*σ*_*eff*_	2.0 / 1.7	3.7 / 2.9	2.4 / 2.3
4D–CBCT	absolute / relative	absolute / relative	absolute / relative
*GM*	0.4 / 0.5	-2.2 / -1.2	-0.6 / -1.4
*Σ*_*eff*_	3.4 / 2.4	5.6 / 4.4	2.3 / 2.2
*σ*_*eff*_	1.9 / 1.7	3.8 / 2.7	2.5 / 2.3
3D– / 4D–CBCT	difference	difference	difference
*GM*	0.3	0.1	-0.4
*Σ*_*eff*_	1.2	1.1	1.0
*σ*_*eff*_	1.2	1.6	1.5

**Fig 3:**
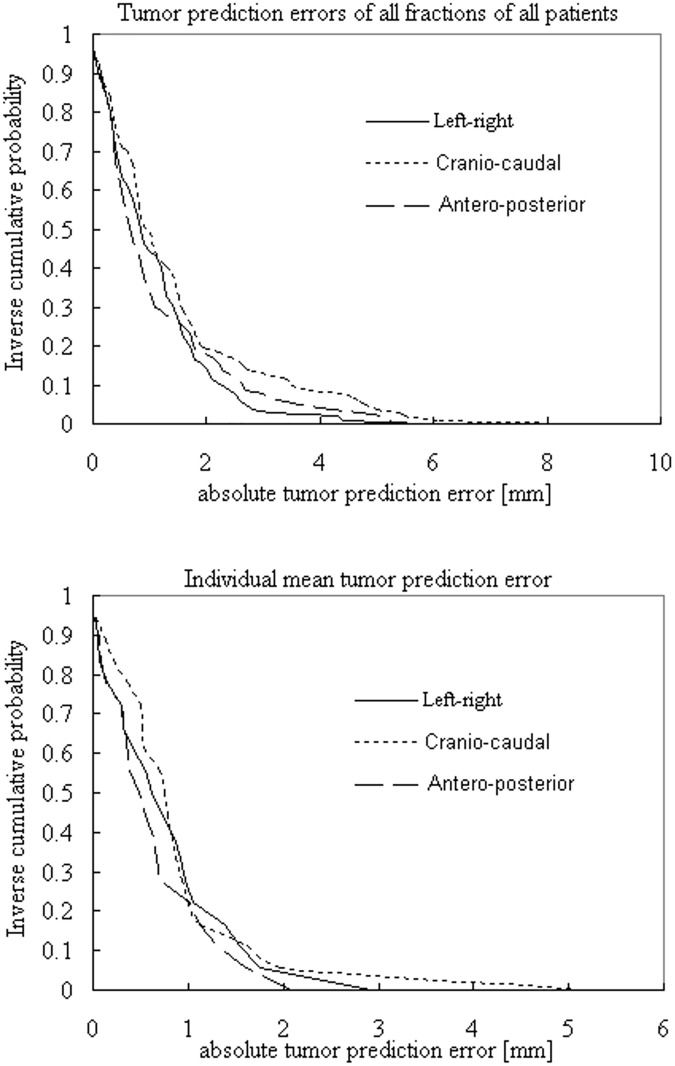
Inverse cumulative probability plots of the absolute tumor prediction errors for all 105 fractions of all 18 patients (top), and for individual means (bottom).

**Fig 4:**
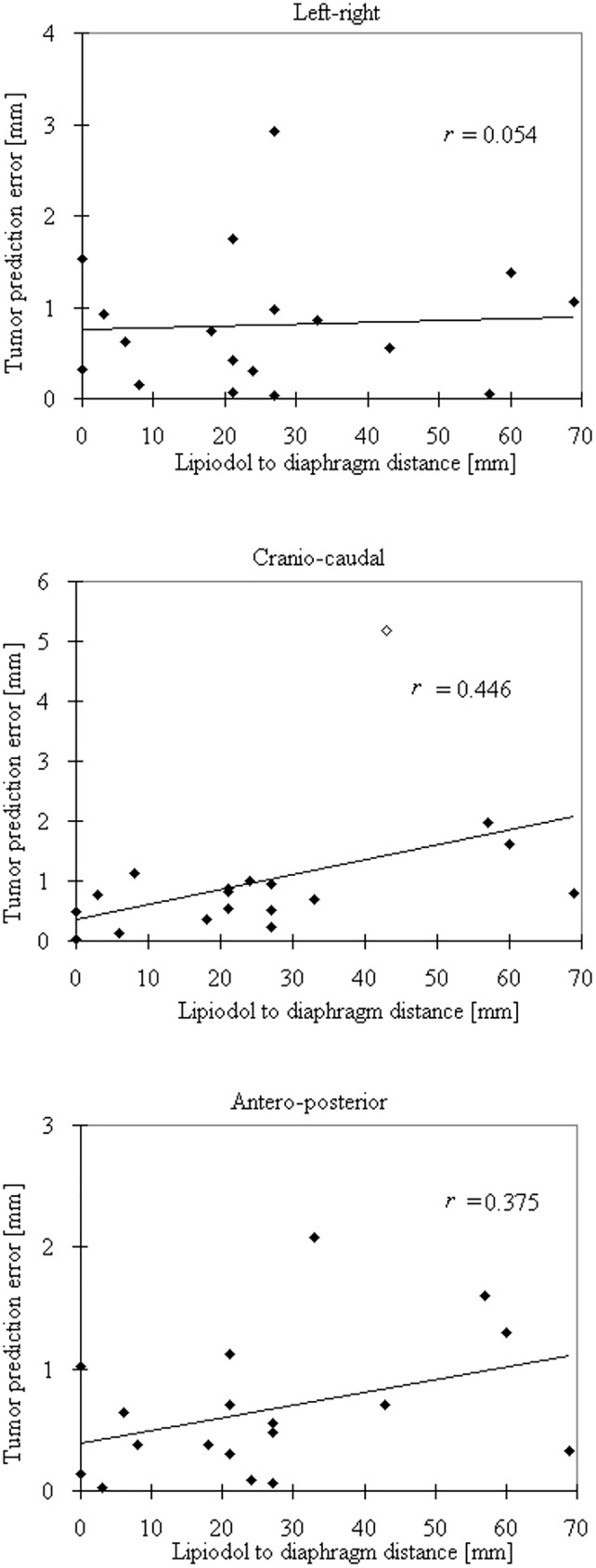
Relationships of lipiodol position between the lipiodol–diaphragm distance and the tumor prediction errors in three cardinal directions. Solid lines are the linear fits with the corresponding Pearson’s correlation coefficients (*r*). The unfilled dot in craniocaudal direction (middle) indicated the outliner.

The absolute tumor prediction errors $\left|{\bar{e}}_{x/y/z}\right|$ are summarized for individual means and for all fractions of all patients in Fig 3. $\left|{\bar{e}}_{x/y/z}\right|$ > 3 and 5 mm was found in one patient only, all occurring in the CC direction, while $\left|{\bar{e}}_{x/y/z}\right|$ > 1 mm occurred in five patients in the LR and AP directions, and in four patients in the CC direction (Fig 4). The LR, CC and AP errors $\left|{\bar{e}}_{x/y/z}\right|$ were not correlated with each other (*p* > 0.05). Borderline statistical significance was found in the correlation between $\left|{\bar{e}}_{y}\right|$ and the tumor–to–diaphragm distance *D* and tumor size, with Pearson’s correlation coefficients (*r*) of 0.45 (*p* = 0.064) and 0.48 (*p* = 0.054), respectively. When the outliner of $\left|{\bar{e}}_{y}\right|$ was removed, its correlation with *D* produced a significant *r* of 0.62 (*p* = 0.008). The variability of $\left|{\bar{e}}_{y}\right|$ (one SD) showed a moderate (*r* = 0.50) but significant (*p* = 0.035) increase with increasing *D*, but not for $\left|{\bar{e}}_{x/z}\right|$. Correlations between $\left|{\bar{e}}_{x/y/z}\right|$ and motion range in each direction were not observed (*p* > 0.05). There were no differences between $\left|{\bar{e}}_{x/y/z}\right|$ and between the variability of $\left|{\bar{e}}_{x/y/z}\right|$.

### Margin assessment

**Fig 5:**
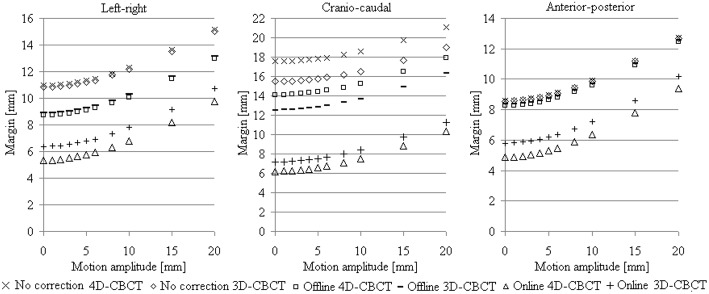
Calculated margins for different treatment approaches. For no image–guidance corrections and offline corrections (i.e. registration to bony anatomy) the margins were calculated using the estimated systematic (*Σ*_*eff*_) and random (*σ*_*eff*_) errors of absolute and relative lipiodol position from 3D– and 4D–CBCT (Table 1). Margins for the online 4D–CBCT using lipiodol registration were obtained assuming ideal soft–tissue tumor correction protocol with zeroed *Σ*_*eff*_ and *σ*_*eff*_, while margins for the online lipiodol–based 3D–CBCT were obtained including*Σ*_*eff*_ and *σ*_*eff*_ due to the offset between 3D– and 4D–CBCT.

Combining tumor delineation uncertainties, 4D–CT /–CBCT registration error of 1 mm (systematic and random), and intra–fractional tumor stability (Case *et al*. [11]) into the van Herk’s margin formula, yields, without any image–guidance correction (i.e., tumor alignment based on external skin marks), the margins as shown in Fig 5. When the variability of lipiodol position from 3D–CBCT entered into the margin recipe, the resulting margins were underestimated by 0.1 (LR) and 2.0 mm (CC) with respect to that calculated from 4D–CBCT for the motion range from 0 to 20mm.

The tumor prediction error resulting from offset between 3D– and reference 4D–CBCT contributed 10.7 (LR), 11.2 (CC), and 10.2 (AP) mm to the PTV for motion up to 20 mm. Using our online lipiodol–guided correction protocol, an additional 1.0 mm margin was required for 3D–CBCT with respect to the reference 4D–CBCT. For comparison to offline bone–alignment correction by 4D–CBCT, the possible gain in margin reduction from online lipiodol–guided correction decreased from 3.4 (LR), 7.9 (CC) and 3.4 mm (AP) using 4D–CBCT to 2.3 (LR), 6.9 (CC) and 2.5 (AP) mm with 3D–CBCT.

## Discussion

The potential advantage of 4D–CBCT may be to mitigate motion–related artifacts and thus potentially allowing more accurate tumor targeting at the time–weighted average position during SBRT. However, systematic comparison with 3D–CBCT was only available for lung tumors so far, but not for liver tumor such as HCC. This may well be due to the fact that without contrast injection, liver tumors are generally not visible because of limited contrast with the parenchyma. As demonstrated by Jones *et al*. [23], intravenous contrast–enhanced CBCT is very sensitive to the acquisition timing, and to our best knowledge has not yet been implemented in routine practice. Contrast enhancement from lipiodol after TACE however may well enable CBCT accuracy assessment for HCC SBRT over time.

Based the analysis of registration errors on clearly visible lipiodol from TACE, this study was for the first time able to assess the tumor positioning results for liver tumors from CBCT with and without motion–correlation. For our cohort of 18 patients, we found that registration errors of 3D– and 4D–CBCT were highly correlated in the three principal motion axes. As a result, the systematic and random errors of these IG–techniques were very similar. Only the systematic error in the CC direction was found to be slightly smaller with 3D–CBCT and the difference was within 1 mm from 4D–CBCT (Table 1). This could be a result of the increased contrast–to–noise ratio due to the increased projections in 3D–CBCT reconstruction (see the following discussions). For both IG–techniques, systematic and random errors and the absolute tumor prediction error over all patients were largest in the CC direction, which also exhibited the most variability (in terms of one SD) among individual means.

Despite the difference in anatomical region, our results were largely consistent with other studies of SBRT for lung tumors. For example, systematic and random differences between 3D– and 4D–CBCT was found to be < 1 mm and the variability between 3D– and 4D–CBCT was largest in the CC direction [7]. However, we found no obvious correlation of the tumor prediction error with the tumor motion, as reported by Sweeney *et al*. [7] for lung tumors. This observation was consistent with the preliminary results of Li *et al*. including ten HCC patients, which concluded that target localization accuracy using the liver contour was found to be unaffected by large breathing amplitudes with 4D–CBCT as seen with 3D–CBCT [24]. A lobe–dependence was observed as the CC tumor prediction errors increased with increasing the tumor–to–diaphragm distance and the dependence reached significance after the removal of one outliner. It was presumed that for tumors located deep at the caudal part of lobe V and VI the accuracy of defining the inferior border of the tumor is subject to increased image artifacts. These may arise from larger offsets from the imaging center and from physiologic motion attributed to an increased uncertainty of the lipiodol position. This was evidenced by the increased variability (SD) of the CC tumor prediction error. It is, however, important to note that the variability of the tumor prediction error did not differ in the cardinal directions, nor did the tumor prediction error itself.

As shown in Fig 5, 3D– and 4D–CBCT resulted in negligible differences in the calculated margins without any image–guidance, which were within 0.1 mm in the LR and AP, and 2.1 mm in the CC direction. The margin size is mainly treatment setup dependent, requiring larger margin up to 13.0 (LR), 17.9 (CC) and 12.4 (AP) mm for offline 4D–CBCT using bone registration compared to 10.7 / 9.8 (LR), 11.2 / 10.4 (CC), 10.2 / 9.4 (AP) mm for online lipiodol–based 3D– / 4D–CBCT. We suggest that for online lipiodol–based registration an additional PTV margin of 1 mm should be considered to offset the GTV’s center of mass on 3D–CBCT with respect to 4D–CBCT for motion up to 20 mm. On the other hand, the 3 mm shift in the caudal direction, indicated by the group mean, was likely due to the finite axial resolution (3 mm) of the MidV images in comparison to the 4D–CBCT with 1 mm axial resolution. The shift may also be a result from basing the treatment planning on CT images corresponding to the MidV instead of the time–weighted average position (MidP). Therefore, the whole PTV should be shifted by 3 and 2 mm in the caudal direction to correct the group means of the lipiodol position for the 3D– and 4D–CBCT setup, respectively.

Although 3D– and 4D–CBCT resulted in similar setup errors in the lipiodol position, 4D–CBCT has the advantage of reducing motion artifacts. Noteworthy though is, that the 3D–CBCT images in this study were synthesized using the projection data that they were acquired with the clinical 4D–CBCT protocol. In fact, the actual clinical 3D–CBCT acquisition protocol takes only 652 projections which were almost half of the 4D–CBCT protocol. As low contrast visibility and structural noise become worse as the number of projections decreases, the image quality of a real 3D–CBCT would be actually worse compared to 4D–CBCT [25]. Nonetheless, the image quality was found to have minimal impact on the registration errors even if the number of projections was reduced to one fourth for the default imaging protocol [25]. The impact of image quality of observer’s variability, however, remains unknown. As a common clinical practice, automatic registration results are subject to validations and further adjustments by specialist clinicians or therapists. Based on the same 3D / 4D–CBCT acquisition and reconstruction protocols as in this study, the concordance between the observers’ (i.e., manual) and the automatic registrations with the liver contour was found to be within 0.75 mm, and the agreement was very high with Krippendorff’s alpha greater than 0.81 in all directions [24]. These preliminary results from Li *et al*. [24] suggested negligible observer’s bias on the final registrations following the automatic 3D and 4D–CBCT registration workflows, given that both images were reconstructed with the same projection data.

Current 4D–CBCT reconstruction algorithms on commercial image–guidance platforms are known to produce sub–optimal image quality without further application of motion–compensation by on–line or prior motion model [15,26]. Great promises of principal component analysis–based prior motion model has been demonstrated by Zhang *et al*. [15], showing much reduced streak artifact due to the sparse projection data from fast acquisition and motion blurring that resulted in improved contrast–to–noise ratio and hence tissue and implanted fiducials visibility. Despite optimal image quality that can be achieved with motion–corrected CBCT, the ability of direct tumor localization remains the most important aspects in SBRT, particular for HCC. We have shown in our previous study that additional margins as large as 5.9 (LR), 10.0 (CC), 2.9 (AP) mm are required to offset of GTV’s center of mass (CoM) from the liver contour’s CoM despite using online 4D–CBCT correction [5], which poses significant limitation to dose escalation to improve the clinical outcomes. The latest IG technology with linac–integrated magnetic resonance imaging (MRI) may be a promising solution for HCC patients without prior lipiodol and intolerable to other invasive procedure of fiducial implantation [27]. A recent clinical trial has been initiated to investigate the potential of MRI–linac in liver tumors [28]. Future studies may be warranted to further investigate the clinical impact of image quality resulting from 4D–CBCT acquisition with and without motion compensation correction and the potential of advanced MRI–guidance compared to 4D–CBCT on target localization for HCC.

## Conclusions

3D–CBCT is an adequate alternative to 4D–CBCT when lipiodol after TACE is used for target localization in free–breathing SBRT of hepatocellular carcinomas. Despite motion artifacts similar safety margins are anticipated with 3D– and 4D–CBCT.
